# Defining a non-eosinophilic inflammatory subtype in COPD: the role of CXCL9 and type 1 immune responses

**DOI:** 10.3389/fimmu.2025.1576849

**Published:** 2025-04-17

**Authors:** Yin Chengsheng, Song Jiacui, Takehiro Hasegawa, Ling Yao, Takami Kondo, Li Huiping

**Affiliations:** ^1^ Department of Respiratory Medicine, Shanghai Pulmonary Hospital, Tongji University School of Medicine, Shanghai, China; ^2^ Research and Development Division, Sysmex R&D Centre Europe GmbH, Hamburg, Germany; ^3^ Application Support, Global Management, Sysmex Corporation, Kobe, Japan; ^4^ Scientific Affairs, Sysmex Corporation, Kobe, Japan

**Keywords:** C-X-C motif chemokine ligand 9 (CXCL9), type 1 immune reactions, chronic obstructive pulmonary disease (COPD), inflammatory pathophysiology, eosinophils

## Abstract

**Background:**

C–X–C motif chemokine ligand 9 (CXCL9) is induced by the interferon-γ response, and its receptor, C–X–C motif chemokine receptor 3, is a well-established marker of T-helper 1 (Th1) cells, which play an essential role in type 1 immune responses. CXCL9 expression is upregulated in patients with interstitial lung disease (ILD), COVID-19, and asthma. Although type 1 inflammation and CD8^+^ T cell activation are considered central to the inflammatory pathophysiology of chronic obstructive pulmonary disease (COPD), the relationship between blood levels of Th1 chemokines and this pathophysiology remains unclear. This study aimed to investigate the relationship between CXCL9 and chronic respiratory diseases.

**Methods:**

We conducted a retrospective cohort study. The serum levels of CXCL9, surfactant protein A (SP-A), Krebs von den Lungen-6 (KL-6), and C-reactive protein (CRP) were analyzed in 165 patients with ILD and COPD. COPD was diagnosed using pulmonary function tests according to the Global Initiative for Chronic Obstructive Lung Disease criteria. Statistical analyses included Fisher’s exact test, Steel–Dwass test, Mann–Whitney *U*, and Wilcoxon test. An unsupervised hierarchical cluster analysis using complete linkage and Euclidean distance was performed for data clustering.

**Results:**

CXCL9 levels were significantly higher in patients with COPD and interstitial ILD than in healthy smokers and non-smokers. The median serum CXCL9 levels in patients with ILD, COPD, healthy smokers, and healthy nonsmokers were 61.6, 69.3, 37.0, and 32.5pg/mL, respectively. CXCL9 levels in patients with COPD significantly correlated with KL-6, SP-A, blood eosinophil ratio, lactate dehydrogenase (LDH), and CRP levels, with correlation coefficients of 0.243, 0.381, 0.225, 0.369, and 0.293, respectively. Additionally, CXCL9 levels were negatively correlated with FEV_1_%. Levels of LDH and KL-6 and the neutrophil ratio were significantly elevated in non-eosinophilic COPD patients with high CXCL9 levels.

**Conclusions:**

Our results highlight the potential role of CXCL9 in the inflammatory pathophysiology of COPD.

## Introduction

The C motif chemokine ligand 9 (CXCL9) is a 12 kDa chemokine composed of 103 amino acids. It is expressed in macrophages, astrocytes, endothelial cells, and epithelial cells, with its expression regulated by the interferon (IFN)-γ response. The receptor for CXCL9, C motif chemokine receptor 3 (CXCR3), is found on T cell subsets, natural killer cells, innate lymphoid cells, macrophages, and B cells. CXCR3 is a well-known marker of T-helper 1 (Th1) cells, which play key roles in type 1 (T1) immune responses. Additionally, IFNs can induce the expression of CXCL10 and CXCL11, which share the same receptor and act similarly to CXCL9 (1).

In a model of cigarette smoke-induced lung inflammation, CXCR3 knockout (KO) mice displayed reduced lung inflammation, characterized by a lower number of CD8^+^ T cells (2) and decreased levels of IFN-γ and CXCR3 ligands, including CXCL9 (3). Elevated serum CXCL9 levels have been reported in patients with asthma, interstitial lung disease (ILD), and COVID-19 infection. In COVID-19 patients, high serum CXCL9 levels are associated with severe conditions such as acute respiratory distress syndrome (ARDS) (4, 5). Furthermore, serum CXCL9 levels are elevated in patients with Stevens–Johnson syndrome and toxic epidermal necrolysis, condition linked to CD8^+^ T cells that produce type 1 pro-inflammatory cytokines (6). Severe COVID-19 symptoms, such as ARDS or kidney failure, are also correlated with elevated CXCL9 levels. Therefore, serum CXCL9 levels are indicative of T1 inflammatory pathophysiology in both *in vitro* and *in vivo* settings.

Chronic obstructive pulmonary disease (COPD) is marked by inflammation in the airways and lung parenchyma. While multiple inflammatory cells contribute to COPD, type 1 inflammation and CD8^+^ T-cell activation are central to its pathogenesis (7). Studies have shown an increased presence of CD8^+^ T cells in the lungs of COPD patients, which correlates with airflow limitation (8, 9). Additionally, elevated levels of Th1-attracting chemokines such as CXCL9, CXCL10, and CXCL11, and their receptor, CXCR3, have been observed in the airways and lung tissue of COPD patients (8). These findings suggest that type 1 inflammatory biomarkers could be valuable for managing COPD. However, recent studies indicate that, similar to asthma, COPD consists of multiple inflammatory subtypes (10). For example, COPD with a type 2 inflammatory phenotype shows a better response to anti-IL-4R inhibitors and inhaled corticosteroids (ICS) (10, 11). In addition to type 1 (T1) and type 2 (T2) inflammation, Th17-type inflammation has also been implicated in COPD, particularly in the induction of autoimmune responses (12). In this study, we investigate the pathology of COPD related to T1 inflammation and examine differences in the type 2 inflammatory phenotype, defined by peripheral blood eosinophil counts.

## Methods

### Patient selection

Patients with preserved specimens and comparable pulmonary function data were recruited from Shanghai Pulmonary Hospital between 2018 and 2021. All samples were collected during a stable period.

A total of 100 patients with COPD were enrolled in this study. Of these, 17 were excluded for the following reasons: incomplete records (n = 2), a time difference > two months between sample collection and the respiratory function test (n = 12), and comorbid infections or inflammatory diseases (n = 3). Ultimately, 83 patients with COPD were included. No patients with asthma-COPD overlap, which may exhibit distinct inflammatory profiles, were included in this study.

In total, 206 patients with ILD were enrolled. Of these, 37 were excluded for the following reasons: incomplete records (n = 17), a time difference > two months between sample collection and the respiratory function test (n = 12), and inflammatory diseases other than autoimmune diseases (n = 8). Finally, 169 patients with ILD were included.

To assess the effect of smoking on neutrophilic airway inflammation, we included 50 healthy smokers (current smokers) and 50 healthy non-smokers (individuals with no history of smoking). No abnormalities were found in chest radiographs of the healthy individuals.

Participants were classified as current smokers (≥10 pack-years), former smokers (quit ≥1 year), or never smokers (<100 lifetime cigarettes).

This study adhered to the principles of the Declaration of Helsinki and was approved by the Medical Research Ethics Committee of Shanghai Pulmonary Hospital (approval number: K17-016). Informed consent was obtained from all patients. Following the Ethical Guidelines for Medical and Health Research Involving Human Subjects, study information was published on the official website, allowing patients to withdraw their consent at any time.

### Study design

In this cross-sectional study, we retrospectively collected samples and clinical data. All serum samples were obtained after patient admission to the hospital. Clinical data were extracted from medical records.

### Data collection

Serum levels of CXCL9, surfactant protein A (SP-A), and Krebs von den Lungen-6 (KL-6) were measured using a fully automatic immunoanalyzer (HISCL™-5000; Sysmex Corp., Hyogo, Japan). C-reactive protein (CRP) levels, hematological results, and biochemical test results were also obtained from the medical records.

### Diagnostic definitions

COPD was diagnosed according to the Global Initiative for Chronic Obstructive Lung Disease (GOLD) guidelines (13). Pulmonary function tests revealed obstructive ventilatory dysfunction, characterized by a post-bronchodilator forced expiratory volume in 1 s to forced vital capacity ratio (FEV_1_/FVC) of <0.7.

The diagnosis of ILD primarily relies on radiological findings, rather than pulmonary function tests. None of the patients enrolled had an FEV1/FVC ratio < 0.7. ILD types included idiopathic pulmonary fibrosis (IPF), idiopathic nonspecific interstitial pneumonia, connective tissue disease-associated interstitial lung disease (CTD-ILD), chronic hypersensitivity pneumonitis (according to the 2022 guidelines), and unclassifiable ILD. Unclassifiable ILD refers to cases that lack a specific diagnosis following a multidisciplinary review of clinical, radiological, and pathological data (14).

### Statistical analysis

Since the data were not normally distributed, descriptive statistics were used and presented as medians with interquartile ranges. Statistical significance was set at p <0.05. Strong, moderate, and weak correlation coefficients were defined as 0.7 ≤ r < 1, 0.3 ≤ r < 0.7, and r < 0.3, respectively. A complete case analysis was performed to address missing data. Fisher’s exact test, Steel–Dwass test, Wilcoxon signed-rank sum test, and Mann–Whitney *U* test were applied using R version 4.4.2 (R project) (15).

## Results

### Demographics and biomarker levels

**Table 1** presents the demographic characteristics of the participants. The study cohort was predominantly male, with 88% of patients having COPD and all healthy smokers being male.

**Table 1:** Demographic and clinical characteristics.

	ILD	COPD	HC smoker	HC nonsmoker	P-value
Age (Year)	62.0 ( 56.0 - 68.0 ) ( 169 )	67.0 ( 62.0 - 74.0 ) ( 83 )	42.5 ( 38.0 - 47.8 ) ( 50 )	36.0 ( 29.3 - 44.8 ) ( 50 )	<0.0001
Sex, Male (%)	111 (65.3)	73 (88.0)	50 (100)	30 (60)	0.0005
FVC% pred	67,4 ( 55.7 - 80.6 ) ( 169 )	70.5 ( 57.4 - 83.5 ) ( 83 )			0.2224
FEV1%pred	70.9 ( 60.2 - 84.0 ) ( 169 )	53.9 ( 37.1 - 67.0 ) ( 83 )			<0.0001
Neutrophil 103 cell/mL	4.3 ( 3.2 - 6.4 ) ( 162 )	4.0 ( 3.2 - 5.1 ) ( 83 )			0.0786
Eosinophil 103 cell/mL	0.1 ( 0.1 - 0.3 ) ( 162 )	0.1 ( 0.1 - 0.3 ) ( 83 )			0.4760
LDH U/L	214.5 ( 175.0 - 270.3 ) ( 162 )	171.0 ( 144.5 - 184.5 ) ( 75 )			<0.0001
CRP mg/dL	4.6 ( 2.3 - 10.8 ) ( 169 )	4.4 ( 3.1 - 7.0 ) ( 78 )			0.8010
IPF, CPFE, CTD-ILD, PAP, other ILD	56,1,29,12,71(33,1,0.6,17.2,7.1,42.5)				
GOLD1,2,3,4 n (%)		12,34,19,18(14.5,44.0,22.9,21.7)			
Smoker, n (%)	40 (23.7)	46 (62.2)	50 (100)	(0)	0.0005
ICS, n (%)		31 (41.9)			
OCS, n (%)	71 (42)	0 (0.0)			

Data are presented as median (interquartile range [IQR]) (n) or n/N (%), where N is the total number of patients with available data. Statistical signific ance between clusters was assessed using the Mann–Whitney U test, Kruskal–Wallis test, or Fisher’s exact test, as appropriate. FVC, forced vital capacity; FEV₁, forced expiratory volume in 1 second; LDH, lactate dehydrogenase; CRP, C-reactive protein; IPF, idiopathic pulmonary fibrosis; CPFE, combined pulmonary fibrosis and emphysema; CTD-ILD, connective tissue disease–associated interstitial lung disease; PAP, pulmo nary alveolar proteinosis; ILD, interstitial lung disease; COPD, chronic obstructive pulmonary disease; ICS, inhaled corticosteroids; OCS, oral cort icosteroids; HC, healthy controls.

The median percentage predicted FVC (%FVC) was 67.4% in patients with ILD and 70.5% in those with COPD. The median predicted forced expiratory volume in 1 s (%FEV_1_) was 70.9% in patients with ILD and 53.9% in those with COPD. According to the GOLD criteria, patients with COPD were classified as follows: 14.5% GOLD I, 44.0% GOLD II, 22.9% GOLD III, and 21.7% GOLD IV.

Among the 169 patients with ILD, 56 (33.0%) had IPF, one (0.6%) had CPFE, 29 (17.2%) had CTD-ILD, 12 (7.1%) had pulmonary alveolar proteinosis, and 71 (42.5%) had unclassified ILD. Additionally, 71 patients (42.0%) had received steroids prior to the first sample collection. Among the patients with COPD, 31 (41.9%) had received ICS.

**Figure 1:**
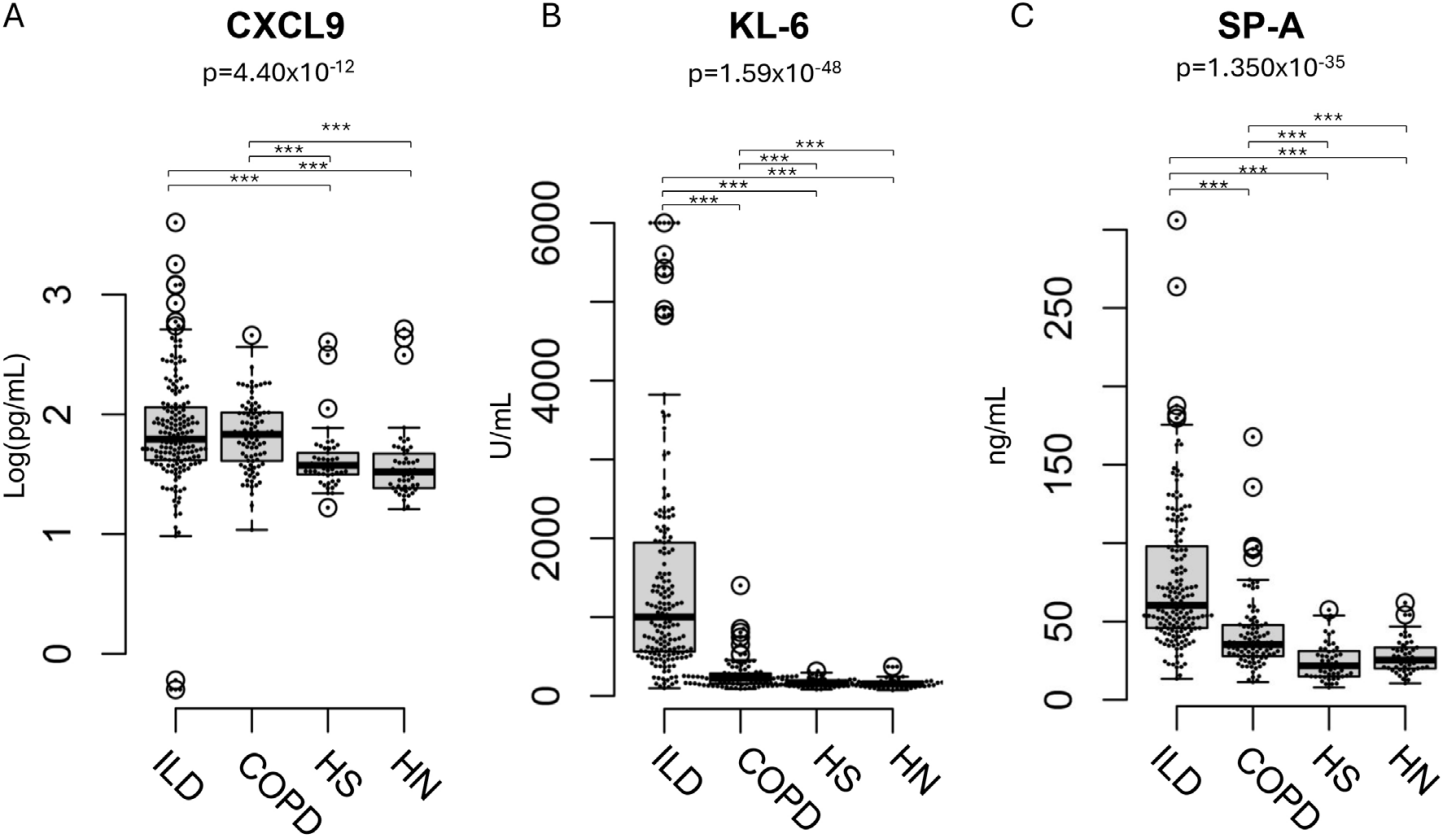
Distribution of CXCL9, KL-6, and SP-A levels ILD patients, COPD patients. **(A)** Distribution of CXCL9, **(B)** KL-6, and **(C)** SP-A levels in patients with interstitial lung disease (ILD), those with chronic obstructive pulmonary disease (COPD), healthy smokers (HS), and healthy non-smokers (HN). The result of the Kruskal-Wallis test is shown in the figure. The *post-hoc* power for CXCL9, KL-6, and SP-A was 0.99, 1.00, and 1.00, respectively. Results are shown as individual data points (circles) with medians (bars) and interquartile ranges (boxes). Outlying values, defined as those more than 1.5 box lengths from the upper or lower edges, are represented as dots outside the whiskers enclosed in circles. The statistical significance between the clusters was calculated using the Steel–Dwass test. ***p < 0.001.

The median serum CXCL9 levels in patients with ILD, those with COPD, healthy smokers, and healthy non-smokers, median serum CXCL9 levels were 61.6, 69.3, 37.0, and 32.5 pg/mL, respectively. Median serum SP-A levels were 60.2, 35.3, 21.7, and 25.4 ng/mL, respectively, and median KL-6 levels were 994.0, 223.0, 162.0, and 151.5 U/mL, respectively (**Table 1**, **Figure 1**). Both ILD and COPD patients had significantly higher serum CXCL9 levels compared to healthy smokers and non-smokers. However, this significance was reduced after adjusting for age differences in the multivariate analysis (**Supplementary Table S1**). Serum levels of these markers were generally lower in ex-smokers with COPD, even though their FEV1/FVC ratios were similar (**Supplementary Figure S1**).

In contrast, no significant differences in serum CXCL9 levels were observed between patients with ILD and those with COPD, nor between healthy smokers and non-smokers.

### Relationship between serum CXCL9 levels and pathophysiology of respiratory diseases

**Figure 2:**
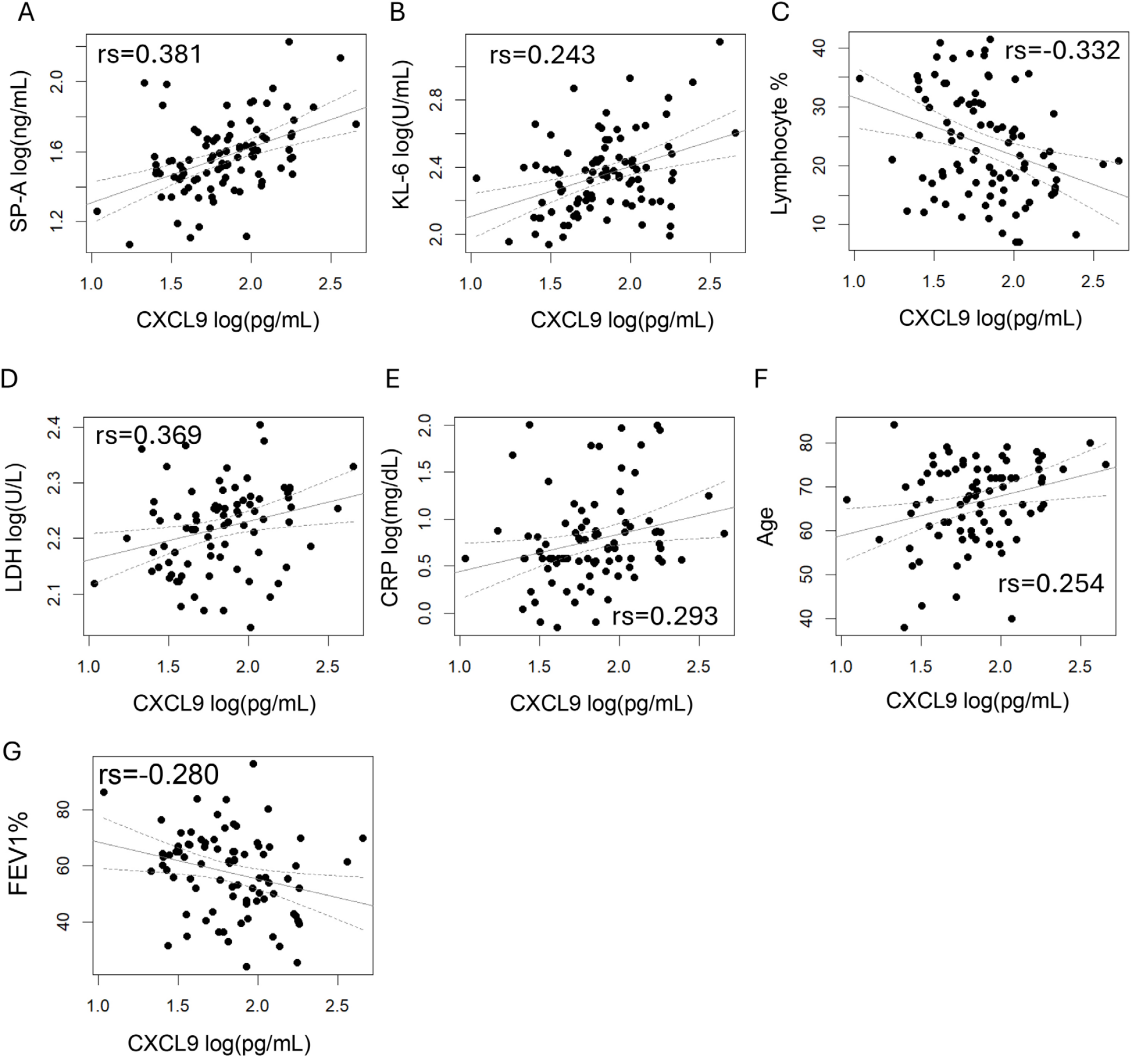
Correlations between pathophysiological parameters and CXCL9 levels in COPD. The correlation coefficients were calculated using Spearman’s correlation analysis. p < 0.05 was considered statistically significant. **(A)** SP-A, **(B)** KL-6, **(C)** Lymphocyte %, **(D)** LDH, **(E)** CRP, **(F)** Age and **(G)** FEV1% showed a significant correlation with CXCL9 levels.

We investigated the correlation between CXCL9 levels and various pathophysiological parameters to better understand its relationship with COPD pathophysiology (**Table 2**; **Figure 2**). In COPD patients, CXCL9 levels were significantly correlated with KL-6 and SP-A levels. The correlation coefficients for SP-A, KL-6, lactate dehydrogenase (LDH), and CRP levels were 0.381, 0.243, 0.369, and 0.293, respectively. CXCL9 levels also showed a negative correlation with FEV_1_% and the ratios of lymphocyte. Multiple regression analyses confirmed significant correlations between CXCL9 levels and SP-A, KL-6, and LDH levels, the ratios of lymphocytes and the ratios of eosinophils (**Supplementary Table S2**).

**Table 2:** Correlations between pathophysiological parameters and CXCL9 levels.

	r_s_	P-value	Adjusted p-value	*Post-hoc* Power
SP-A	0.381	0.000	0.007	0.950
KL-6	0.243	0.027	0.066	0.606
Neutrophil %	0.183	0.098	0.166	0.384
Lymphocyte %	-0.332	0.002	0.012	0.873
Monocyte %	0.190	0.087	0.164	0.409
Eosinophil %	0.225	0.041	0.086	0.540
Basophil %	0.033	0.769	0.872	0.060
LDH	0.369	0.001	0.010	0.936
CRP	0.293	0.009	0.039	0.774
Age	0.270	0.014	0.039	0.701
FVC% Pred.	-0.025	0.824	0.876	0.056
FEV1. %Pred	-0.110	0.322	0.421	0.168
FEV1/FVC	-0.280	0.010	0.035	0.735
DLco	-0.187	0.146	0.226	0.230
SpO2	-0.123	0.271	0.383	0.199
PaO2	-0.079	0.483	0.586	0.109
PaCO2	0.020	0.856	0.856	0.054

Correlation coefficients were calculated using Spearman’s correlation analysis, and the p-values were adjusted using the Benjamini-Hochberg method.

### COPD and inflammatory marker levels

**Figure 3:**
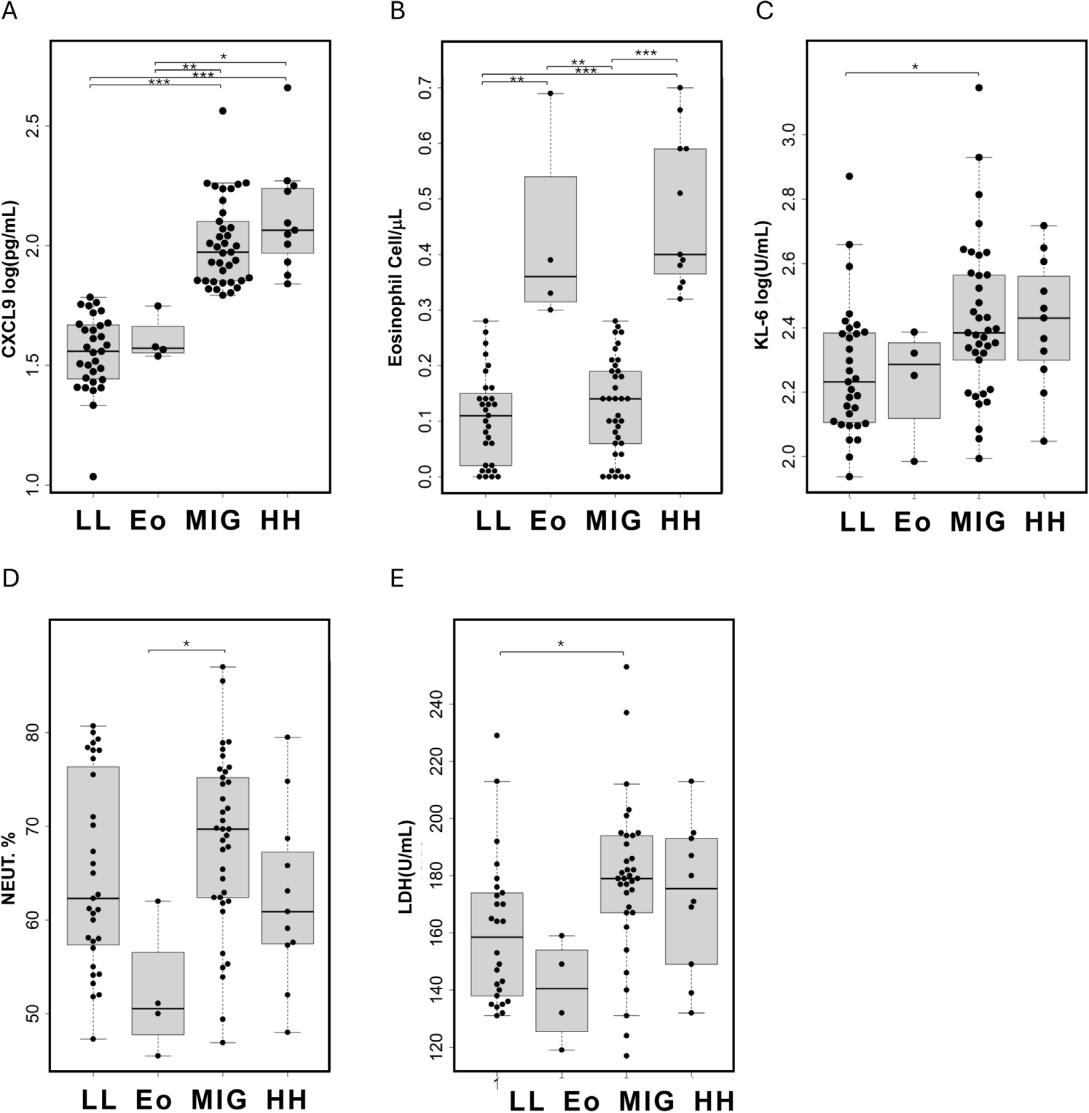
Comparison of patient characteristics in the four identified class based on serum CXCL9 and eosinophil count levels in patients with COPD. LL, CXCL9 low and eosinophil count low; Eo, only eosinophil high; MIG, CXCL9 high; HH, CXCL9 and eosinophil count high. **(A)** Serum CXCL9 levels, **(B)** blood eosinophil counts, **(C)** serum KL-6 levels, **(D)** blood neutrophil ratios, and **(E)** serum LDH levels are presented as individual data points (circles) with medians (horizontal bars) and interquartile ranges (boxes). Outlying values, defined as those more than 1.5 box lengths from the upper or lower edges, are represented as dots outside the whiskers enclosed in circles. The statistical significance between the classes were calculated using the Steel–Dwass test. *p < 0.05, **p < 0.01, and ***p < 0.001.

A subtype of COPD characterized by blood eosinophil counts exceeding 300 cells/μL has been suggested, with evidence indicating a better response to T2 anti-inflammatory treatments such as ICS or dupilumab. Since CXCL9 levels were significantly correlated with the eosinophil ratio, we analyzed the differences in pathophysiological representation between CXCL9 and eosinophils (**Figure 3**, **Supplementary Table S3**).

Patients were classified into four subtypes based on their CXCL9 levels and eosinophil counts. The cutoff value for CXCL9 was set at 61.15 pg/mL, corresponding to the 90th percentile value in healthy individuals (smokers and non-smokers), and the cutoff for eosinophil count was 300 cells/μL. Among these patients, 31 were negative for both CXCL9 and eosinophils (LL), four were positive only for eosinophils (Eo), 32 were positive only for CXCL9 (MIG), and 11 were positive for both CXCL9 and eosinophils (HH).

KL-6 and LDH levels were significantly higher in the MIG group compared to the LL group, and the neutrophil ratio was significantly higher in the MIG group than in the Eo group. Although the median FEV_1_% was lower in the MIG and HH groups, the difference was not statistically significant. In multiple logistic regression analysis, CXCL9 levels were significantly associated with KL-6 and LDH levels, after adjusting for age, sex, eosinophil count, smoking status, and ICS treatment (**Supplementary Table S4**).

## Discussion

This study examined the role of CXCL9 in COPD pathophysiology by measuring its serum levels and evaluating its potential as a specific marker of T1-driven inflammation.

A large-scale study on COPD severity distribution reported that the proportions of patients with GOLD stages I–IV were 10.1%, 41.7%, 34.5%, and 13.6%, respectively (16). In contrast, our study found that 22.9% of patients were in GOLD III and 21.7% in GOLD IV, indicating a slight skew toward more severe cases. Additionally, we observed elevated KL-6 and SP-A levels in COPD patients compared to healthy controls (HC), though these levels remained below the established cutoffs levels for ILD (17, 18).

CXCL9 levels were compared among patients with ILD or COPD, healthy smokers, and healthy non-smokers (**Figure 1**). At the 90th percentile, CXCL9 levels in healthy smokers and non-smokers were 59.0 pg/mL and 62.4 pg/mL, respectively. These levels were slightly higher than those reported in previous Japanese studies using the same reagent with HISCL™, where the 95th percentile was 39.0 pg/mL (1). Significant age differences were noted between the HC and patient groups. While our previous research indicated that CXCL9 levels in healthy individuals increased slightly with age, the differences were minimal compared to those observed in disease conditions. Among HC over 60 years, CXCL9 levels reached 20.5 pg/mL (interquartile range 18.0–22.3 pg/mL), remaining below the 95th percentile and significantly lower than those seen in ILD or COPD patients (1). Although a substantial age difference masked the variation in CXCL9 levels during multivariate analysis (**Supplementary Table 1**), age was not a predominant factor compared to CXCL9 levels in pathophysiological analysis (**Supplementary Tables 2 and 3**). Therefore, age distribution differences between patients and HC are unlikely to affect the clinical significance of CXCL9 levels.

COPD is characterized by chronic inflammation of the airways and lung parenchyma, leading to progressive airflow limitation. Various inflammatory cells, such as eosinophils, neutrophils, CD8^+^ T-cells, Th1 cells, and alveolar macrophages, interact to create distinct inflammatory profiles in individual patients (2, 8, 9). In addition to T1 inflammation, pro-inflammatory cytokines such as TNF-α, IL-6, and IL-17 play crucial roles in COPD pathogenesis. TNF-α promotes neutrophilic inflammation and alveolar destruction, IL-6 contributes to systemic inflammation and COPD-associated comorbidities, and IL-17 drives neutrophilic recruitment and airway remodeling, contributing to chronic inflammation and steroid resistance (12, 19). Although cigarette smoking is the primary risk factor for COPD, chronic exposure to biomass-burning smoke also independently induces distinct systemic inflammation characterized by elevated cytokines such as IL-6, IL-8, and IP-10 (20). Additionally, genetic susceptibility (e.g., PiS variant) may further enhance COPD risk among non-smoking populations exposed to biomass smoke may further enhance COPD risk among non-smoking populations exposed to biomass smoke (21). Patient classification based on specific inflammatory pathophysiology is essential for precision medicine in COPD. Recently, a subtype called eosinophilic COPD was identified based on blood eosinophil counts. Studies suggest that these patients respond well to ICS and dupilumab, a specific inhibitor of IL-4Ra (10, 11). However, biomarkers for other inflammatory COPD subtypes remain poorly understood (10).

In COPD patients, monocytes exhibit enhanced chemotactic responses, attracting Tc1 and Th1 cells via the CXCR3 receptor (22). Increased infiltration of CXCR3-expressing and CD8+ T cells has been observed in COPD lung tissue (19). Furthermore, CD4+ Th1, Th17, and CD8+ T cells are elevated in COPD lungs and strongly correlate with disease severity (12). CD8^+^ T cells in COPD also demonstrate higher perforin and toll-like receptor expression, with an increased capacity to induce IFN-γ and TNF-α, both of which act as CXCL9 inducers (23).

CXCL9, a chemokine induced by IFN-γ, has been shown to correlate significantly between serum and bronchoalveolar lavage fluid in patients with chronic hypersensitivity pneumonitis and autoimmune disease-related ILD (24, 25). These findings suggest that serum CXCL9 levels reflect Th1/Tc1-driven inflammation and its role in disease progression.

In this study, serum CXCL9 levels were significantly elevated in COPD and ILD and correlated with KL-6, SP-A, CRP, LDH, the blood eosinophil ratio, and FEV_1_% (**Figure 2**). However, the correlations between CXCL9 and KL-6, SP-A, and LDH levels were weak, preventing the establishment of a direct causal relationship.

KL-6, a marker of alveolar epithelial cell injury, is elevated in ILD and acute lung injury. It has also been detected at higher levels in COPD patients, correlating with disease severity and poor prognosis (26, 27). Similarly, SP-A is increased in COPD serum, correlating with reduced lung function and increased inflammation, making it a potential biomarker for disease severity (28, 29). LDH, a cytoplasmic enzyme released into the bloodstream due to cell membrane disruption, is commonly used as a marker of cell injury and death (30). Its activity is elevated in asthma, chronic cough, and COPD (26, 31, 32).

These findings suggest that elevated serum KL-6 and LDH levels may indicate severe tissue damage in COPD, whereas CXCL9 levels may reflect the underlying inflammatory processes driving this damage.

Further analysis classified COPD patients into four groups based on blood eosinophil ratios and CXCL9 levels. KL-6 and LDH levels were elevated in both the MIG and H/H groups, indicating significant respiratory damage. In the MIG group, neutrophil ratios were significantly higher, suggesting that neutrophilic inflammation, distinct from eosinophilic COPD, was the primary contributor to respiratory damage. Additionally, recent research has linked COPD-associated autoimmune responses primarily to type 1 inflammation. This suggests that MIG-type patients may have an increased risk of developing autoimmune diseases (12).

This retrospective study has several limitations. First, it included a relatively small observational cohort, with a limited number of patients with eosinophilic COPD. Additionally, COPD disease activity scores, such as the Modified Medical Research Council Dyspnea Scale or COPD Assessment Test, were not collected. Given its cross-sectional nature, this study also could not infer causality. Furthermore, detailed exposure data to environmental pollutants other than smoking, such as biomass-burning smoke or ambient air pollution, were not systematically collected, which might constitute a potential confounder influencing the inflammatory profiles observed. Long-term, longitudinal studies are needed to clarify the relationship between CXCL9 levels and disease progression. While our findings provide substantial evidence regarding the role of T1 inflammation in COPD, further research is required to explore this aspect in greater depth and validate our results in broader contexts.

In conclusion, this study demonstrated that serum CXCL9 level serves as a distinct T1 inflammatory marker of COPD, independent of T2 inflammation, which is defined by eosinophilic inflammation. These findings suggest that the incorporation of CXCL9 into clinical assessments could improve COPD phenotyping and open new avenues for targeted anti-inflammatory therapies.

## Data Availability

The data that support the findings of this study are available from Shanghai Pulmonary Hospital, but restrictions apply to the availability of these data, which were used under license for the current study and are not publicly available. However, data are available from the authors upon reasonable request and with permission from the Medical Research Ethics Committee of Shanghai Pulmonary Hospital.
