# Supplementary material for: Defining a non-eosinophilic inflammatory subtype in COPD: the role of CXCL9 and type 1 immune responses
Source: Front Immunol. 2025 Apr 17;16:1576849. doi: 10.3389/fimmu.2025.1576849 (PMC12043484; doi:10.3389/fimmu.2025.1576849)
Supplement: Supplementary file 1 [file Presentation1.pptx]

## Slide 1
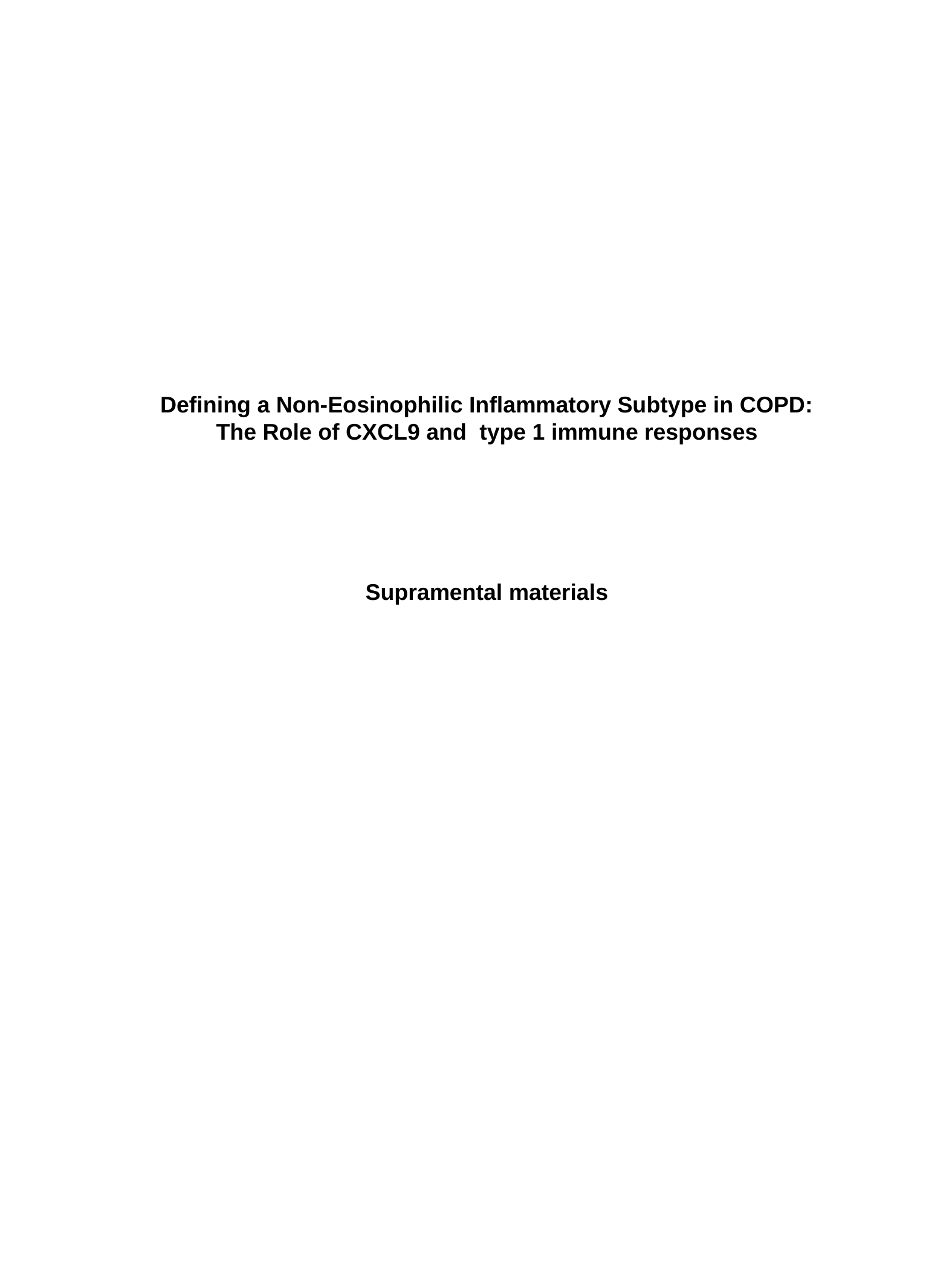

Defining a Non-Eosinophilic Inflammatory Subtype in COPD: The Role of CXCL9 and type 1 immune responses
Supramental materials

## Slide 2
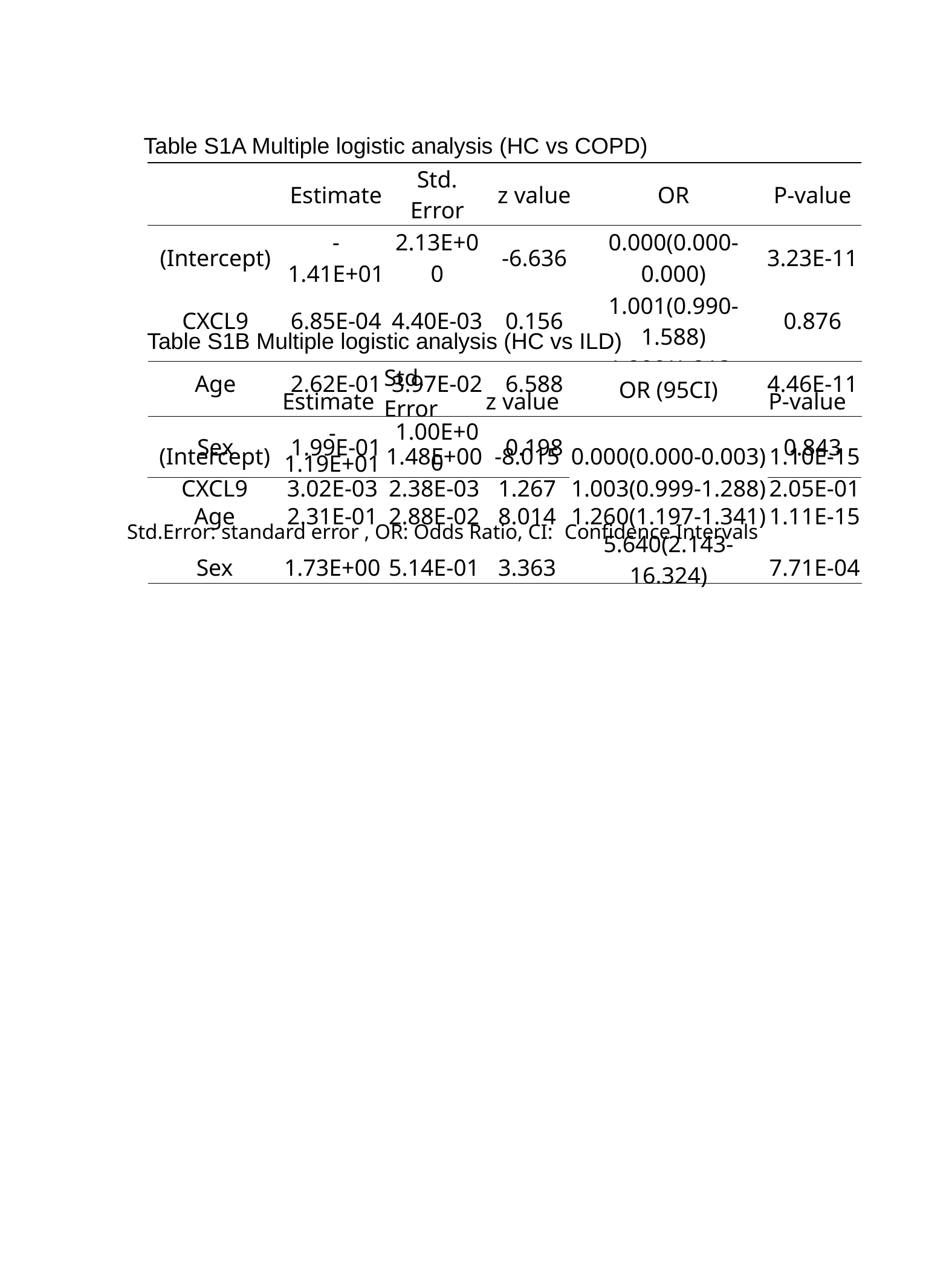

Table S1A Multiple logistic analysis (HC vs COPD)
| | Estimate | Std. Error | z value | OR | P-value |
| --- | --- | --- | --- | --- | --- |
| (Intercept) | -1.41E+01 | 2.13E+00 | -6.636 | 0.000(0.000-0.000) | 3.23E-11 |
| CXCL9 | 6.85E-04 | 4.40E-03 | 0.156 | 1.001(0.990-1.588) | 0.876 |
| Age | 2.62E-01 | 3.97E-02 | 6.588 | 1.299(1.213-1.421) | 4.46E-11 |
| Sex | 1.99E-01 | 1.00E+00 | 0.198 | 1.220(0.164-8.434) | 0.843 |
Table S1B Multiple logistic analysis (HC vs ILD)
| | Estimate | Std. Error | z value | OR (95CI) | P-value |
| --- | --- | --- | --- | --- | --- |
| (Intercept) | -1.19E+01 | 1.48E+00 | -8.015 | 0.000(0.000-0.003) | 1.10E-15 |
| CXCL9 | 3.02E-03 | 2.38E-03 | 1.267 | 1.003(0.999-1.288) | 2.05E-01 |
| Age | 2.31E-01 | 2.88E-02 | 8.014 | 1.260(1.197-1.341) | 1.11E-15 |
| Sex | 1.73E+00 | 5.14E-01 | 3.363 | 5.640(2.143-16.324) | 7.71E-04 |
| | | | | | |
Std.Error: standard error , OR: Odds Ratio, CI: Confidence Intervals

## Slide 3
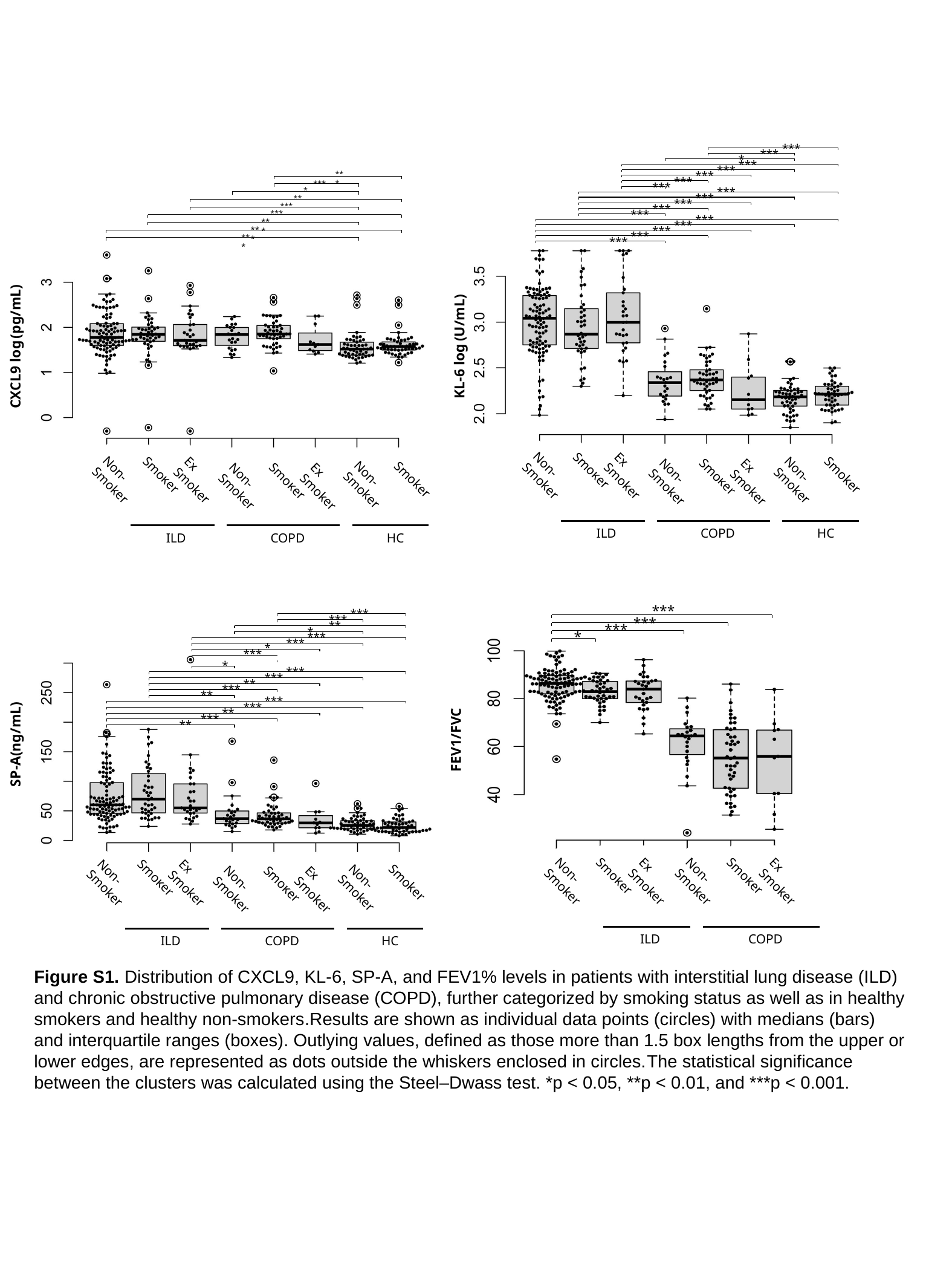

***
***
*
***
***
***
***
***
***
***
***
***
***
***
***
***
***
***
***
***
*
**
***
***
***
***
***
CXCL9 log (pg/mL)
KL-6 log (U/mL)
Smoker
Ex Smoker
Smoker
Non-Smoker
Smoker
Ex Smoker
Non-Smoker
Non-Smoker
COPD
HC
ILD
Smoker
Ex Smoker
Smoker
Non-Smoker
Smoker
Ex Smoker
Non-Smoker
Non-Smoker
COPD
HC
ILD
***
***
***
*
***
***
**
*
***
***
*
***
*
***
***
**
***
**
***
***
**
***
**
SP-A(ng/mL)
Smoker
Ex Smoker
Smoker
Non-Smoker
Smoker
Ex Smoker
Non-Smoker
Non-Smoker
COPD
HC
ILD
FEV1/FVC
Smoker
Smoker
Ex Smoker
Ex Smoker
Non-Smoker
Non-Smoker
COPD
ILD
Figure S1. Distribution of CXCL9, KL-6, SP-A, and FEV1% levels in patients with interstitial lung disease (ILD) and chronic obstructive pulmonary disease (COPD), further categorized by smoking status as well as in healthy smokers and healthy non-smokers.Results are shown as individual data points (circles) with medians (bars) and interquartile ranges (boxes). Outlying values, defined as those more than 1.5 box lengths from the upper or lower edges, are represented as dots outside the whiskers enclosed in circles.The statistical significance between the clusters was calculated using the Steel–Dwass test. *p < 0.05, **p < 0.01, and ***p < 0.001.

## Slide 4
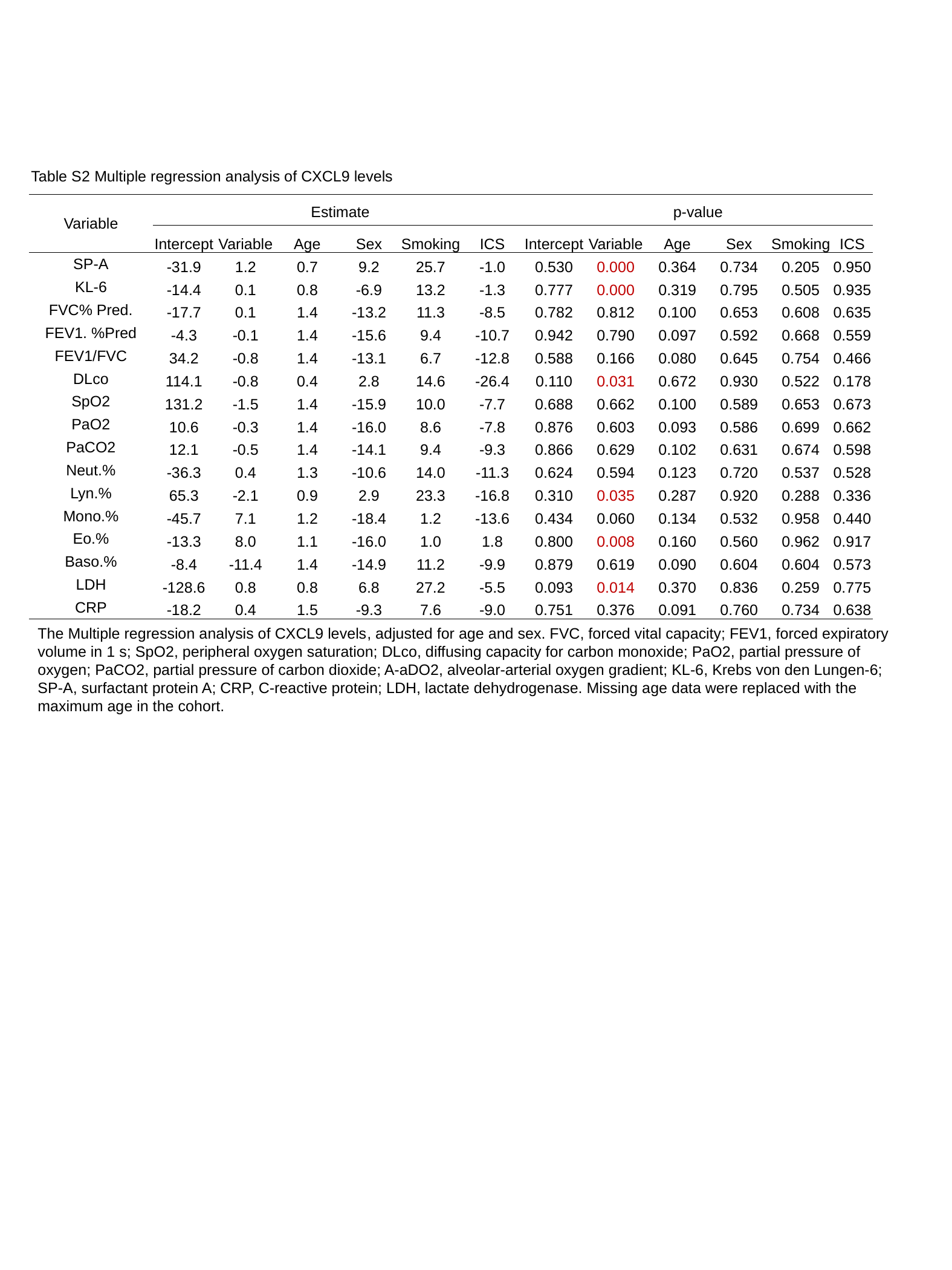

Table S2 Multiple regression analysis of CXCL9 levels
| Variable | Estimate | | | | | | p-value | | | | | |
| --- | --- | --- | --- | --- | --- | --- | --- | --- | --- | --- | --- | --- |
| | Intercept | Variable | Age | Sex | Smoking | ICS | Intercept | Variable | Age | Sex | Smoking | ICS |
| SP-A | -31.9 | 1.2 | 0.7 | 9.2 | 25.7 | -1.0 | 0.530 | 0.000 | 0.364 | 0.734 | 0.205 | 0.950 |
| KL-6 | -14.4 | 0.1 | 0.8 | -6.9 | 13.2 | -1.3 | 0.777 | 0.000 | 0.319 | 0.795 | 0.505 | 0.935 |
| FVC% Pred. | -17.7 | 0.1 | 1.4 | -13.2 | 11.3 | -8.5 | 0.782 | 0.812 | 0.100 | 0.653 | 0.608 | 0.635 |
| FEV1. %Pred | -4.3 | -0.1 | 1.4 | -15.6 | 9.4 | -10.7 | 0.942 | 0.790 | 0.097 | 0.592 | 0.668 | 0.559 |
| FEV1/FVC | 34.2 | -0.8 | 1.4 | -13.1 | 6.7 | -12.8 | 0.588 | 0.166 | 0.080 | 0.645 | 0.754 | 0.466 |
| DLco | 114.1 | -0.8 | 0.4 | 2.8 | 14.6 | -26.4 | 0.110 | 0.031 | 0.672 | 0.930 | 0.522 | 0.178 |
| SpO2 | 131.2 | -1.5 | 1.4 | -15.9 | 10.0 | -7.7 | 0.688 | 0.662 | 0.100 | 0.589 | 0.653 | 0.673 |
| PaO2 | 10.6 | -0.3 | 1.4 | -16.0 | 8.6 | -7.8 | 0.876 | 0.603 | 0.093 | 0.586 | 0.699 | 0.662 |
| PaCO2 | 12.1 | -0.5 | 1.4 | -14.1 | 9.4 | -9.3 | 0.866 | 0.629 | 0.102 | 0.631 | 0.674 | 0.598 |
| Neut.% | -36.3 | 0.4 | 1.3 | -10.6 | 14.0 | -11.3 | 0.624 | 0.594 | 0.123 | 0.720 | 0.537 | 0.528 |
| Lyn.% | 65.3 | -2.1 | 0.9 | 2.9 | 23.3 | -16.8 | 0.310 | 0.035 | 0.287 | 0.920 | 0.288 | 0.336 |
| Mono.% | -45.7 | 7.1 | 1.2 | -18.4 | 1.2 | -13.6 | 0.434 | 0.060 | 0.134 | 0.532 | 0.958 | 0.440 |
| Eo.% | -13.3 | 8.0 | 1.1 | -16.0 | 1.0 | 1.8 | 0.800 | 0.008 | 0.160 | 0.560 | 0.962 | 0.917 |
| Baso.% | -8.4 | -11.4 | 1.4 | -14.9 | 11.2 | -9.9 | 0.879 | 0.619 | 0.090 | 0.604 | 0.604 | 0.573 |
| LDH | -128.6 | 0.8 | 0.8 | 6.8 | 27.2 | -5.5 | 0.093 | 0.014 | 0.370 | 0.836 | 0.259 | 0.775 |
| CRP | -18.2 | 0.4 | 1.5 | -9.3 | 7.6 | -9.0 | 0.751 | 0.376 | 0.091 | 0.760 | 0.734 | 0.638 |
The Multiple regression analysis of CXCL9 levels, adjusted for age and sex. FVC, forced vital capacity; FEV1, forced expiratory volume in 1 s; SpO2, peripheral oxygen saturation; DLco, diffusing capacity for carbon monoxide; PaO2, partial pressure of oxygen; PaCO2, partial pressure of carbon dioxide; A-aDO2, alveolar-arterial oxygen gradient; KL-6, Krebs von den Lungen-6; SP-A, surfactant protein A; CRP, C-reactive protein; LDH, lactate dehydrogenase. Missing age data were replaced with the maximum age in the cohort.

## Slide 5
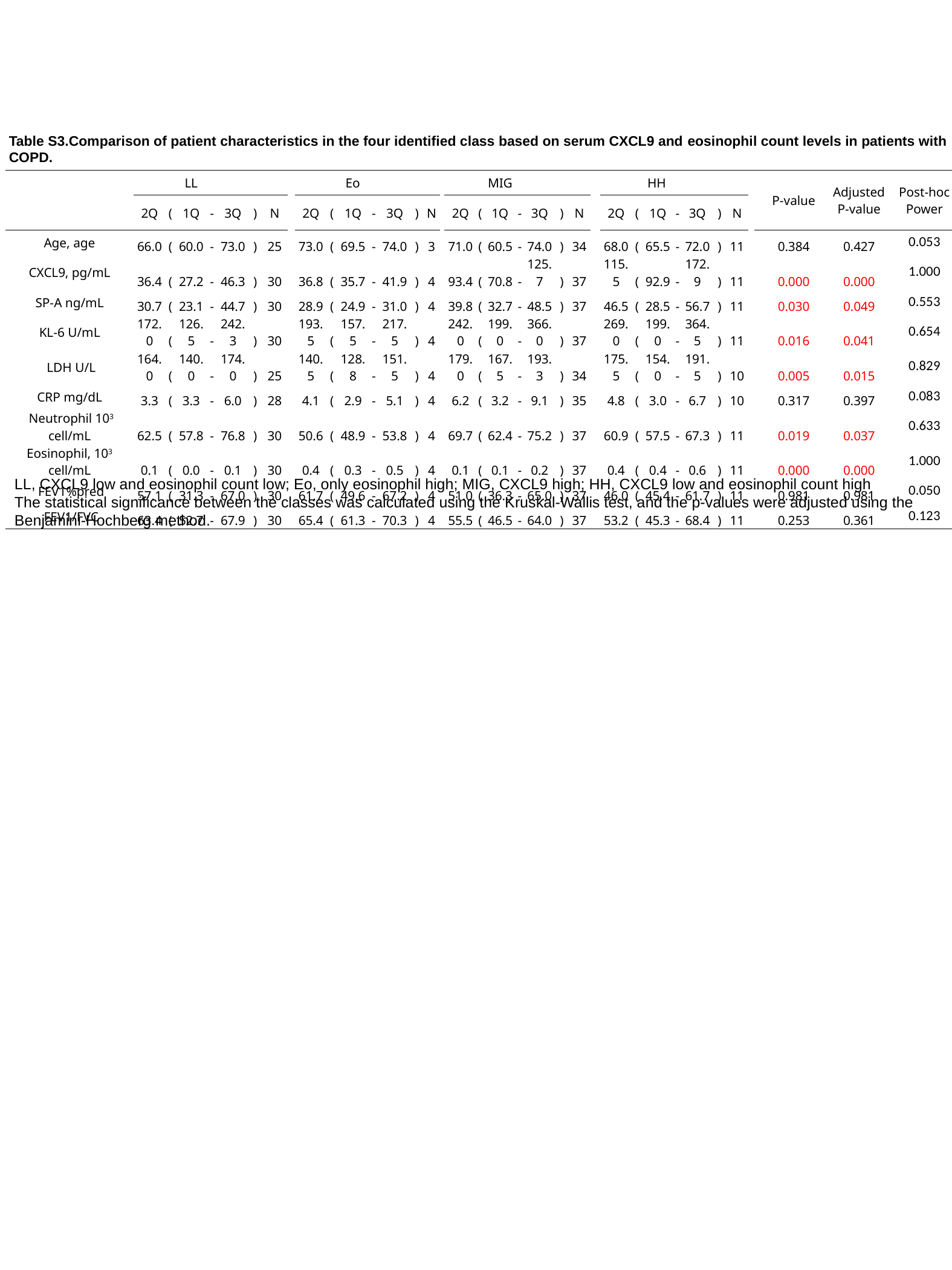

Table S3.Comparison of patient characteristics in the four identified class based on serum CXCL9 and eosinophil count levels in patients with COPD.
| | LL | | | | | | | | Eo | | | | | | | | MIG | | | | | | | | HH | | | | | | | | | P-value | Adjusted P-value | Post-hoc Power |
| --- | --- | --- | --- | --- | --- | --- | --- | --- | --- | --- | --- | --- | --- | --- | --- | --- | --- | --- | --- | --- | --- | --- | --- | --- | --- | --- | --- | --- | --- | --- | --- | --- | --- | --- | --- | --- |
| | 2Q | ( | 1Q | - | 3Q | ) | N | | 2Q | ( | 1Q | - | 3Q | ) | N | | 2Q | ( | 1Q | - | 3Q | ) | N | | 2Q | ( | 1Q | - | 3Q | ) | N | | | | | |
| Age, age | 66.0 | ( | 60.0 | - | 73.0 | ) | 25 | | 73.0 | ( | 69.5 | - | 74.0 | ) | 3 | | 71.0 | ( | 60.5 | - | 74.0 | ) | 34 | | 68.0 | ( | 65.5 | - | 72.0 | ) | 11 | | | 0.384 | 0.427 | 0.053 |
| CXCL9, pg/mL | 36.4 | ( | 27.2 | - | 46.3 | ) | 30 | | 36.8 | ( | 35.7 | - | 41.9 | ) | 4 | | 93.4 | ( | 70.8 | - | 125.7 | ) | 37 | | 115.5 | ( | 92.9 | - | 172.9 | ) | 11 | | | 0.000 | 0.000 | 1.000 |
| SP-A ng/mL | 30.7 | ( | 23.1 | - | 44.7 | ) | 30 | | 28.9 | ( | 24.9 | - | 31.0 | ) | 4 | | 39.8 | ( | 32.7 | - | 48.5 | ) | 37 | | 46.5 | ( | 28.5 | - | 56.7 | ) | 11 | | | 0.030 | 0.049 | 0.553 |
| KL-6 U/mL | 172.0 | ( | 126.5 | - | 242.3 | ) | 30 | | 193.5 | ( | 157.5 | - | 217.5 | ) | 4 | | 242.0 | ( | 199.0 | - | 366.0 | ) | 37 | | 269.0 | ( | 199.0 | - | 364.5 | ) | 11 | | | 0.016 | 0.041 | 0.654 |
| LDH U/L | 164.0 | ( | 140.0 | - | 174.0 | ) | 25 | | 140.5 | ( | 128.8 | - | 151.5 | ) | 4 | | 179.0 | ( | 167.5 | - | 193.3 | ) | 34 | | 175.5 | ( | 154.0 | - | 191.5 | ) | 10 | | | 0.005 | 0.015 | 0.829 |
| CRP mg/dL | 3.3 | ( | 3.3 | - | 6.0 | ) | 28 | | 4.1 | ( | 2.9 | - | 5.1 | ) | 4 | | 6.2 | ( | 3.2 | - | 9.1 | ) | 35 | | 4.8 | ( | 3.0 | - | 6.7 | ) | 10 | | | 0.317 | 0.397 | 0.083 |
| Neutrophil 103 cell/mL | 62.5 | ( | 57.8 | - | 76.8 | ) | 30 | | 50.6 | ( | 48.9 | - | 53.8 | ) | 4 | | 69.7 | ( | 62.4 | - | 75.2 | ) | 37 | | 60.9 | ( | 57.5 | - | 67.3 | ) | 11 | | | 0.019 | 0.037 | 0.633 |
| Eosinophil, 103 cell/mL | 0.1 | ( | 0.0 | - | 0.1 | ) | 30 | | 0.4 | ( | 0.3 | - | 0.5 | ) | 4 | | 0.1 | ( | 0.1 | - | 0.2 | ) | 37 | | 0.4 | ( | 0.4 | - | 0.6 | ) | 11 | | | 0.000 | 0.000 | 1.000 |
| FEV1%pred | 57.1 | ( | 31.3 | - | 67.0 | ) | 30 | | 61.7 | ( | 49.6 | - | 67.2 | ) | 4 | | 51.0 | ( | 36.3 | - | 65.0 | ) | 37 | | 46.0 | ( | 45.4 | - | 61.7 | ) | 11 | | | 0.981 | 0.981 | 0.050 |
| FEV1/FVC | 63.4 | ( | 52.7 | - | 67.9 | ) | 30 | | 65.4 | ( | 61.3 | - | 70.3 | ) | 4 | | 55.5 | ( | 46.5 | - | 64.0 | ) | 37 | | 53.2 | ( | 45.3 | - | 68.4 | ) | 11 | | | 0.253 | 0.361 | 0.123 |
LL, CXCL9 low and eosinophil count low; Eo, only eosinophil high; MIG, CXCL9 high; HH, CXCL9 low and eosinophil count high
The statistical significance between the classes was calculated using the Kruskal-Wallis test, and the p-values were adjusted using the Benjamini-Hochberg method.

## Slide 6
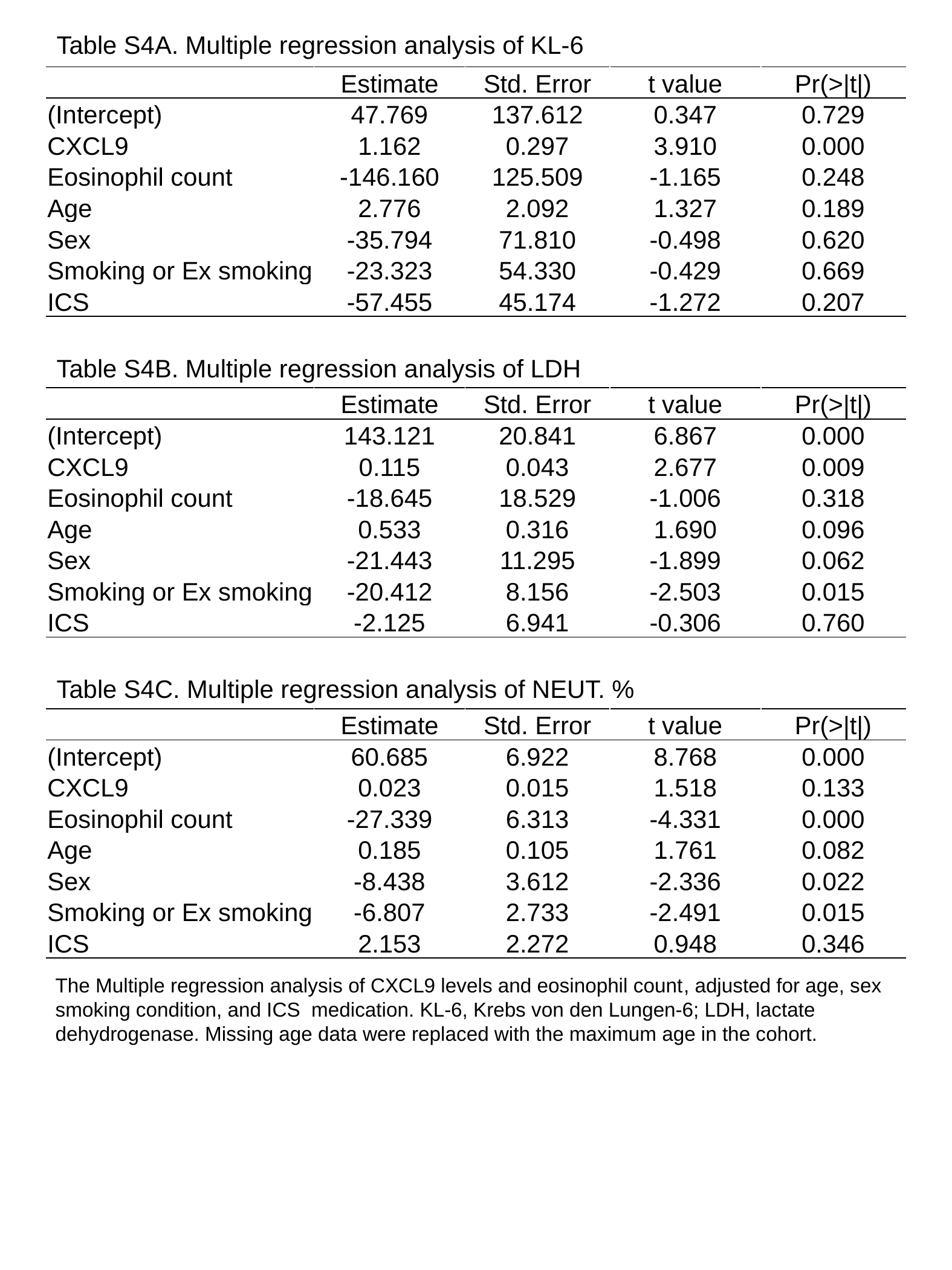

| Table S4A. Multiple regression analysis of KL-6 | | | | |
| --- | --- | --- | --- | --- |
| | Estimate | Std. Error | t value | Pr(>|t|) |
| (Intercept) | 47.769 | 137.612 | 0.347 | 0.729 |
| CXCL9 | 1.162 | 0.297 | 3.910 | 0.000 |
| Eosinophil count | -146.160 | 125.509 | -1.165 | 0.248 |
| Age | 2.776 | 2.092 | 1.327 | 0.189 |
| Sex | -35.794 | 71.810 | -0.498 | 0.620 |
| Smoking or Ex smoking | -23.323 | 54.330 | -0.429 | 0.669 |
| ICS | -57.455 | 45.174 | -1.272 | 0.207 |
| | | | | |
| Table S4B. Multiple regression analysis of LDH | | | | |
| | Estimate | Std. Error | t value | Pr(>|t|) |
| (Intercept) | 143.121 | 20.841 | 6.867 | 0.000 |
| CXCL9 | 0.115 | 0.043 | 2.677 | 0.009 |
| Eosinophil count | -18.645 | 18.529 | -1.006 | 0.318 |
| Age | 0.533 | 0.316 | 1.690 | 0.096 |
| Sex | -21.443 | 11.295 | -1.899 | 0.062 |
| Smoking or Ex smoking | -20.412 | 8.156 | -2.503 | 0.015 |
| ICS | -2.125 | 6.941 | -0.306 | 0.760 |
| | | | | |
| Table S4C. Multiple regression analysis of NEUT. % | | | | |
| | Estimate | Std. Error | t value | Pr(>|t|) |
| (Intercept) | 60.685 | 6.922 | 8.768 | 0.000 |
| CXCL9 | 0.023 | 0.015 | 1.518 | 0.133 |
| Eosinophil count | -27.339 | 6.313 | -4.331 | 0.000 |
| Age | 0.185 | 0.105 | 1.761 | 0.082 |
| Sex | -8.438 | 3.612 | -2.336 | 0.022 |
| Smoking or Ex smoking | -6.807 | 2.733 | -2.491 | 0.015 |
| ICS | 2.153 | 2.272 | 0.948 | 0.346 |
The Multiple regression analysis of CXCL9 levels and eosinophil count, adjusted for age, sex smoking condition, and ICS medication. KL-6, Krebs von den Lungen-6; LDH, lactate dehydrogenase. Missing age data were replaced with the maximum age in the cohort.
